# Sugar Alcohols, Caries Incidence, and Remineralization of Caries Lesions: A Literature Review

**DOI:** 10.1155/2010/981072

**Published:** 2010-01-05

**Authors:** Kauko K. Mäkinen

**Affiliations:** Institute of Dentistry, University of Turku, Lemminkäisenkatu 2, 20520 Turku, Finland

## Abstract

Remineralization of minor enamel defects is a normal physiological process that is well known to clinicians and researchers in dentistry and oral biology. This process can be facilitated by various dietary and oral hygiene procedures and may also concern dentin caries lesions. Dental caries is reversible if detected and treated sufficiently early. Habitual use of xylitol, a sugar alcohol of the pentitol type, can be associated with significant reduction in caries incidence and with tooth remineralization. Other dietary polyols that can remarkably lower the incidence of caries include erythritol which is a tetritol-type alditol. Based on known molecular parameters of simple dietary alditols, it is conceivable to predict that their efficacy in caries prevention will follow the homologous series, that is, that the number of OH-groups present in the alditol molecule will determine the efficacy as follows: erythritol ≥ xylitol > sorbitol. The possible difference between erythritol and xylitol must be confirmed in future clinical trials.

## 1. Introduction

The ability of human saliva to reharden acid-softened enamel was definitively first reported by Head in 1912 [1]. His report was not appreciated owing to the then-exiguous understanding of the chemical mechanisms that underlie innate host defence reactions, including the natural rehardening of caries lesions. Later, Koulourides, Pigman, and their contemporaries started to pay attention to remineralization of dental enamel by saliva [2–4]. Other earlier works are referred to in the latter references. More recent reviews and treatises were provided, for example, by the Demineralization/Remineralization Working Group [5] and by Kashket [6]. Several researchers, including Leach et al. [7] and Edgar [8] have emphasized the role that diet can play in tooth remineralization.

Rehardening of deep dentin caries lesions is not commonly encountered in most industrialized countries because dentists normally treat the teeth long before an initial lesion reaches the dentin caries level. It is well known, however, that also untreated, open dentin caries lesions can undergo a rehardening process, provided that the microbiological and chemical environment of the carious tooth changes remarkably. Such changes can be occasioned by a significant improvement of oral hygiene and remarkable changes in dietary habits. The common clinical rule is, of course, that dentin caries should always require operative treatment in order to save the tooth. The everyday reality can, however, be very different in many juvenile populations, especially in developing countries that lack the necessary dental resources. On the other hand, comment has also been made about remineralization of advanced dentinal lesions in nonindustrialized societies where restorations are not commonly completed by a dentist [9]. Furthermore, hardening, darkening, and continued service for many years by remineralized teeth have been noted [10]. 

Clinical studies and laboratory experiments have shown that the usage of certain dietary sugar alcohols (polyols), notably xylitol and sorbitol, can be associated with rehardening of artificial and genuine caries lesions. It is surprising that previous remineralization reviews have not mentioned even a single scientific study in this field, that is, sugar alcohol-associated remineralization. Earlier reviews have ignored the potential that sugar alcohols, especially xylitol, can provide in the planning of caries prevention strategies that may lead to remineralization of caries lesions. Consequently, the aim of this treatise is (1) to summarize the clinical caries trials on xylitol, (2) to examine those chemical and biologic features of sugar alcohols that are assumed to play a role in caries prevention and tooth remineralization, (3) to summarize the roles that innate salivary factors can play in physiologic remineralization, and (4) to review results obtained in clinical and laboratory remineralization studies with sugar alcohols. The majority of remineralization-associated investigations have been carried out with xylitol and sorbitol. Several reviews have dealt with caries limitation and sugar alcohols (*vide infra*). Consequently, the present treatise focuses mainly on tooth remineralization. The cariostatic potential of erythritol, a sugar alcohol of the tetritol type, will also be discussed.

## 2. Clinical Caries Trials on Sugar Alcohols

The first dental caries studies on xylitol began at the Institute of Dentistry at the University of Turku in Finland in late 1969. The results of these studies showed that consumption of xylitol reduced the growth of dental plaque in participating dental students by up to 50% compared with use of sucrose, *D*-glucose, or *D*-fructose [11, 12]. Based on these observations, a two-year clinical caries study and a one-year chewing-gum trial (collectively called the Turku Sugar Studies) were set up in 1972-1973 [13]. The results showed that xylitol consumption was associated with an impressive caries reduction and prompted other researchers to repeat the Turku studies. Accordingly, a newly formed “xylitol concept” was presented in 1975 to the world scientific dental health community for purposes of expansion and verification. The confirmatory rounds of testing carried out during the next 30 years showed that the most important original claims of the dental efficacy of xylitol were verified by independent researchers in long-term clinical trials which were carried out under greatly varying and challenging conditions. Nineteen clinical trials and most of the nearly 300 short-term oral biologic laboratory studies on xylitol have been reviewed and commented [14–29]. 

The presently available information on clinical trials on the caries-limiting effects of xylitol (reviewed in the above publications) is summarized in Table 1. Several trials also investigated sorbitol. The accumulated data of these trials have in part constituted the rationale behind current public endorsements of xylitol worldwide [30, 31]. The clinical efficacy of xylitol in caries prevention was further discussed and reviewed at the U.S. National Institutes of Health Consensus Conference in 2001 [26, 32]. 

**Figure 1:**
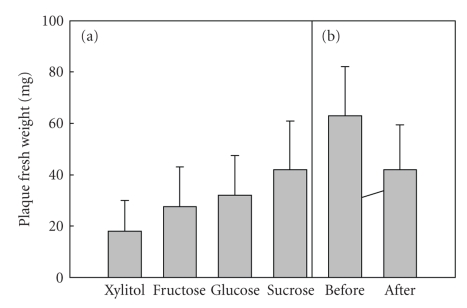
“How it all began”: a pioneering plaque assessment study carried out in 1970 (a). Effect of dietary carbohydrates and xylitol on the growth of dental plaque after consumption of the shown sweeteners for four days (while the subjects refrained from oral hygiene), mainly in coffee or tea, and in the form of hard candies [11]. The consumption level of each sweetener was about 20 g per day and per subject. The values shown are means ± S.D. of fresh weight of plaque collected from all available tooth surfaces. (b) Inverse relationship between plaque fresh weight and its protein content. Twelve test subjects used xylitol chewing gum five times a day over a period of one month. Plaque from all available surfaces was collected following a 2-day no-oral-hygiene period. Consumption level of xylitol per day and per subject was 6.7 g. Xylitol consumption was associated with reduced plaque mass while the protein content of plaque simultaneously rose from 1.1 ± 0.2 mg to 1.4 ± 0.2 mg per mL of plaque suspension (straight line). Protein and nitrogen analyses should not be claimed to accurately determine the amount of dental plaque in clinical studies involving sugar alcohols.

The two plaque studies [11, 12], mentioned above, deserve attention since they can justifiably be regarded as pioneers of the subsequent wealth of dental investigations on xylitol. Figure 1(a) shows a rendering of the “how-it-all-began” plaque study published in 1971. Four-day use of xylitol as a sweetener in subjects' diet resulted in about 50% reduction in plaque compared with the use of sucrose. The strong plaque mass-reducing effect of xylitol along with supportive biochemical effects observed in plaque in 1970-1971 [11, 12] generated first the Turku Sugar Studies [13] and next all other xylitol caries trials shown in Table 1. Figure 1(b) additionally reveals a particular oral biologic feature of dental plaque exposed to xylitol: the increase of nitrogen- and protein-associated metabolism in plaque—although the plaque volume, mass, adhesiveness, and cariogenicity simultaneously decrease, and its alkalinity increases. The reason for emphasizing this plaque feature stems from the exploitation of protein or nitrogen determination as a single plaque assessment procedure by some authors who have been unaware of the biochemical effects of xylitol on plaque. This situation has led to erroneous conclusions on the true changes in plaque virulence as affected by xylitol [20]. In other words, plaque protein analysis should not be used as the sole (and decisive) analytical method to measure the effect of sugar alcohols on plaque.

## 3. “Utility Value” of Polyol Strategies in Caries Prevention

Although the clinical efficacy of sugar alcohols in caries prevention is incontestable (Table 1), it is important to evaluate their true utility value assessed by independent meta-analyses and cost/benefit evaluations. A study by Deshpande and Jadad [33] on the impact of polyol-containing chewing gums on dental caries concluded that “research evidence supports using polyol-containing chewing gum as part of normal oral hygiene to prevent dental caries”. The mean “preventive fraction” (PR; with 95% confidence interval) for the use of xylitol, xylitol-sorbitol blend, and sorbitol was 58.66% (35.42–81.90), 52.82% (39.64–66.00), and 20.01% (12.74–27.27), respectively. For the sorbitol-mannitol blend, the value of PR was 10.71% (−20.50–41.93), which is statistically insignificant. The appraisal of Mickenautsch et al. [34] arrived at an almost identical conclusion. One of the studies evaluated was that of Machiulskiene et al. [35], carried out in Lithuania. It was not possible to deduce from the data of Deshpande and Jadad [33] and Mickenautsch et al. [34] whether the subsequent “rectifying” paper of Hayes [36] had been considered; Hayes determined that xylitol gum (among the several gums tested in the Lithuanian study) was the only gum that lowered the DMFS increment compared with the no-gum group after three years. The original authors [35] of the Lithuanian study had failed to recognize the potential of xylitol gum in their own clinical trial. Other public health evaluations [29, 37] have supported the above contentions on the potential that dental-protective chewing gums can provide in oral health interventions.

Further support for the utility value of xylitol interventions was provided by Milgrom et al. [38–40], who were among the first to determine the dose and frequency response of xylitol usage on mutans streptococci growth. A commendable effort was made by Milgrom et al. [39], who examined all xylitol products available on the United States market. Another practical achievement was the use of a xylitol-containing gummy bear snack as a means to reduce the growth of *S. mutans*/*sobrinus* levels [41]. The efficacy of xylitol in a dentifrice has been demonstrated in several studies [42–45], and in a pacifier as a slow-release mechanism in infants [46]. Further support for the practicality of xylitol usage has been obtained from its suggested synergistic effects with fluoride [47, 48], its use in combination with chlorhexidine [49, 50], as an alternative to fissure sealing [51], and as a sweetener in hard candies [52]. The practicality of the use of sorbitol may be somewhat affected by its low sweetness, the adaptation of oral streptococci to sorbitol [53, 54], and its customary support of the growth of dental plaque and mutans streptococci.

Use of xylitol in caries limitation has been investigated from the point of view of public health [55–57]. These surveys provided information on factual usage of xylitol as a caries-limiting agent. They reviewed public endorsements and use of xylitol for caries prevention programs worldwide [30]. It is significant that researchers have emphasized the role that xylitol-containing chewing gums could potentially have as a preventive measure in public health [58]. Basic research began to provide meaningful explanations for the observed xylitol effects about twenty years ago [15, 59, 60].

## 4. Literature Searches on Tooth Remineralization

Online literature searches provide interesting historical views into tooth remineralization research. Table 2 shows a typical PubMed search using the program's own MeSH vocabulary, whose term for the search was “tooth remineralization”. The number of references per year presumed during the 1980s the current reference rate of more than 40 per year. These figures tend to change over time, however, since more detailed searches retroactively add new references to the database. The Science Citation Index gave slightly different figures. Selecting “enamel rehardening” as the key word resulted in 16 references through the ages, the oldest one from 1964, while simple “remineralization” yielded a total of 2268 references (as of December 17, 2008), the oldest one from 1910, but this figure includes a large number of nondental references. The increase in the number of dental remineralization papers reflects general acceptance within the relevant clinical and scientific circles that the remineralization of caries lesions is a normal physiologic repair process that can also be aided by various dietary measures.

## 5. Nonspecific Xylitol Effects versus Specific Ones

The use of xylitol in food and oral hygiene products has been found to result in significant caries reduction (Table 1 and the above references). Remineralization and rehardening are terms that have most often been employed to describe the type of caries arrest demonstrated in clinical xylitol programs and laboratory studies. Several studies suggest that xylitol can exert specific effects on dental caries not shown by hexitols. Authors who have opposed the existence of specific xylitol-associated effects in caries reduction have contended that caries reduction observed after xylitol use can simply be explained in terms of the following passive xylitol effects.

- Involvement of mere salivary effects, that is, the increase in salivation regularly associated with the consumption of sweet items, constitutes the only reason that explains the clinical observations made with xylitol in caries prevention studies.
- Mere partial removal of a caries-inducive agent (sugar, notably sucrose) from the diet and substituting it with an essentially non-fermentable sweetener (xylitol) explains the observed caries reduction. In other words, in the presence of xylitol the cariogenic organisms are merely deprived of their normal growth substrate. The growth of dental plaque and the progression of caries will reduce only as a result of partial removal of the cariogenic challenge.

The above passive xylitol effects naturally constitute an important cornerstone in xylitol-associated caries limitation and would even as such fully justify the promotion of xylitol as a caries-reducing agent. The scientific review papers and professional evaluations thus far published have not denied this fact. Scientific literature is, however, replete with findings that also support the involvement of active, specific xylitol effects that operate even in the presence of fermentable hexose-based carbohydrates, that is, in situations where a strong cariogenic challenge is present. In the Turku Sugar Studies [13] sucrose and xylitol gums differed significantly from each other in their caries-limiting ability in a situation where the salivary involvement (i.e., the chewing effect) was regarded as similar in both study cohorts. The Belize studies [61] and the preceding animal experiments [62–64] also support the idea of specific xylitol effects; xylitol was found to limit dental caries even in the presence of a strong cariogenic challenge and was more effective than sorbitol. 

Shyu and Hsu [63] showed in rats that alditols differed significantly in their caries-reducing potential: xylitol caused the lowest caries scores (86% reduction). *D*-Mannitol reduced caries by 70%, sorbitol by 48%, and plain diet by 39% (compared with sucrose added to the plain diet; all sweeteners were tested at a 10% level). This study also reflects a problem that plagues most comparative dental trials on dietary sweeteners: the use of percentage levels. When 10% of either xylitol (mol. wt. 152.1), sorbitol (182.2), mannitol (182.2), or sucrose (342.3) is expressed in molarities (connoting the number of molecules present in solution), the true chemical concentrations differ significantly, that is, 0.657 M for xylitol, 0.549 M for the hexitols, and 0.292 M for sucrose. Comparing 10% erythritol (122.1) with 10% sucrose reveals even more remarkable differences in true chemical concentrations: 0.819 M versus 0.292 M. Critical appraisal of clinical studies should thus be conducted by considering the above discrepancy in expressing concentrations. However, the conclusions of Shyu and Hsu [63] were not remarkably affected by the above criticism.

Some of the physicochemical properties of xylitol discussed below will further elucidate the complex scientific background that is assumed to lie behind the clinical effects reported in literature. All dental xylitol studies have not, however, reached positive clinical and oral biologic findings. Long-term field experience has shown that in most cases failures in demonstrating such effects can be explained in terms of the following features of the studies in question.

- Use of caries-resistant study cohorts or cohorts with extremely low caries experience.
- Use of too-small study cohorts.
- Use of too-low concentrations of xylitol.
- Use of too-short intervention.
- Use of too-short or too-infrequent exposure to xylitol.
- Simultaneous use of other caries-limiting agents and strategies (such as fluorides).
- Use of too-insensitive analytical procedures.
- Use of a single analytical procedure to assess oral biologic parameters (such as plaque growth).

A recommended practice is to use 6 to 7 g of xylitol daily, preferably in 3 to 5 separate episodes. Regarding oral biologic measurements (such as plaque growth, microbiology, and the chemical composition of saliva and plaque), experiments lasting from a few days to several months or even years, have been implemented. Regarding dental caries outcomes, trials that last several years are recommended. A particular dilemma has indeed been occasioned by studies that have leaned toward a single plaque assessment procedure (such as protein determination, which can lead to erroneous conclusions). In plaque studies, it is advantageous to rely on simultaneous gravimetric, planimetric (before and after, using disclosing dyes and colour photography), clinical (plaque index), bacteriologic, chemical, and enzyme measurements. 

Among enzyme determinations, an analysis of the combined invertase (EC 3.2.1.26) and sucrase (EC 3.2.1.48) activities have turned out promising. (The numbers shown refer to the Enzyme Commission's classification.) Consumption of a xylitol diet is normally associated with significantly decreased whole saliva and plaque invertase-sucrase activity levels [13], suggesting diminished sucrose-splitting capacity with concomitantly reduced acid production. Reduced dextranase (EC 3.2.1.11) activity of dental plaque may also result from xylitol consumption [13]; this enzyme causes endohydrolysis of 1,6-*α*-*D*-glucosidic linkages in dextran, whose levels in plaque are normally high after sugar consumption. Similarly, strongly reduced salivary *α*-amylase (EC 3.2.1.1) activity has been found in xylitol-consuming subjects [13]. The activity levels of *α*-*L*-fucosidase (EC 3.2.1.51) may in turn increase during xylitol consumption (possibly resulting from increased hydrolysis of the named fucoside linkages present in salivary glycoproteins; this process may be important in the formation of the acquired tooth pellicle). All of the above glycosidases can be regarded as markers of plaque metabolism. Similarly, certain proteinase and aminopeptidase activities (which are normally increased in plaque after xylitol use) can be regarded as suitable markers of plaque biochemistry. The above changes in enzyme activity can normally be encountered when analyzing plaque and plaque extracellular fluid (with the exception of *α*-amylase, which is derived from glandular saliva), indicating that the origin of those activities is predominantly in the oral microbiota. The proteinase, aminopeptidase, and *α*-fucosidase activities increased most likely because plaque microorganisms were deprived of their preferred growth substrates (hexose-based sugars) and converted their energy-yielding enzyme activities towards the proteins and glycoproteins present in saliva. This resulted in an overall increase in plaque and saliva nitrogen metabolism. The reduction in *α*-amylase, dextranase, and invertase/sucrase activities can in turn be interpreted as resulting from the lowering of the sucrose levels in the subjects' diet during xylitol regimen.

## 6. Chemical Features of Sugar Alcohols That Can be Associated with Remineralization

The term “sugar alcohol” refers in chemical colloquialism to the reduction products of “sugars”, indicating that all oxygen atoms present in a simple sugar alcohol molecule are in the form of hydroxyl groups. The terms “polyol” and “polyhydric alcohol” in turn refer to chemical compounds that contain three or more hydroxyl groups. All sugar alcohols are polyols. The polyols can be divided into acyclic compounds (alditols or glycitols, which can be regarded as true sugar alcohols) and cyclic polyols. Examples of the former are erythritol, xylitol, and *D*-glucitol (sorbitol), while *myo*-inositol serves as an example of cyclic polyols. The most important dietary sugar alcohols that will be discussed in this treatise include the four-, five-, and six-carbon members of the homologous alditol series, that is, erythritol, xylitol, sorbitol, and related polyols. These molecules are based on a single monosaccharide skeleton. Among disaccharide sugar alcohols, maltitol (derived from maltose), lactitol (derived from lactose), and palatinit (equimolar mixture of *α*-*D*-glucopyranosyl-1,6-sorbitol and *α*-*D*-glucopyranosyl-1,6-*D*-mannitol) have received attention in nutritional and special medical uses. Complex, long-chain polyols have been manufactured for various food uses by hydrogenation of starch hydrolysates. Such products (hydrogenated starch syrups) often contain varying amounts of simpler polyols (such as sorbitol, maltitol, and trimeric and even higher homologues) as by-products.

According to carbohydrate nomenclature, it is permissible to use the name adonitol for ribitol, arabitol for arabinitol, sorbitol for *D*-glucitol, and dulcitol for galactitol. The name mannitol requires *D* or *L*. Names without *D* and *L* include erythritol, xylitol, ribitol, sorbitol, dulcitol, lactitol, and maltitol (only those polyhydric alcohols are mentioned that will be discussed in the present text). Erythritol (1,2,3,4-tetrahydroxybutane) has appeared in texts as *meso*-erythritol and *i*-erythritol; *meso* in this case stands for optical inactivity owing to internal compensation.

The simple alditols are crystalline substances varying in taste from faintly sweet (galactitol) to very sweet (erythritol and xylitol, which are almost isosweet with sucrose). Several alditols, notably erythritol and xylitol, exert relatively strong negative heat of solution, a physiochemical property that in practice is reflected in the perception of a “cooling effect” in the mouth as crystalline alditol dissolves in saliva; the energy which is required in the dissolution process is taken from the environment, producing a cooling effect. Of the hexitols, sorbitol and *D*-mannitol show specific optical rotation for sodium *D* line, [*α*_D_], while galactitol is optically inactive. Xylitol, ribitol, and erythritol are also optically inactive. Historic and various evolutionary and chemical aspects of sugar alcohols have been discussed in other contexts [17, 18, 84–87]. 

For the purpose of the present discussion, it is necessary to review some of the common sugar alcohol properties as follows.

**Figure 2:**
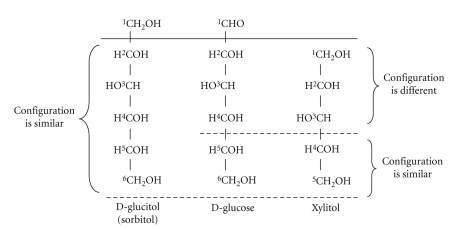
Relationship between the structural configurations of sorbitol (*D*-glucitol), *D*-glucose, and xylitol. The molecular configurations of sorbitol and glucose are relatively similar. Hence, sorbitol can be called a “glucose-polyol”. The configuration of xylitol (a “non-glucose polyol”) markedly differs from the two other configurations. The close similarity of sorbitol with glucose partly explains its plaque-promoting and mutans streptococci-stimulating effects.

**Figure 3:**
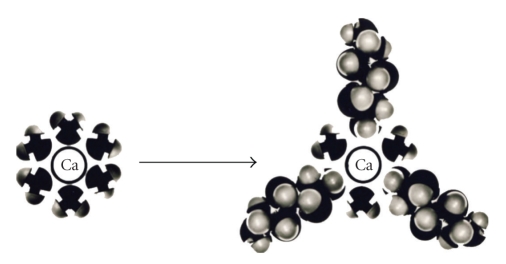
A simplified presentation of the competition between water and xylitol molecules for Ca, assumed to play a role in environments involving whole-mouth saliva and plaque fluid. Here, Ca has interacted with six water molecules which constitute the primary hydration layer of the metal ion (the actual number of water molecules surrounding the spherical Ca ion may vary from 4 to 12). The resulting new hydration layer consists of water molecules and xylitol molecules. This leads to stabilization of the salivary Ca phosphate systems [14, 18]. Reproduced with permission [14].

(a) *Absence of a reducing group*. The absence of a reducing carbonyl group in the alditol molecules makes them chemically somewhat less reactive than the corresponding aldoses and ketoses. Some sugar alcohols can thus avoid those chemical reactions that normally make many dietary hexose-based sugars acidogenic and cariogenic in dental plaque. Xylitol, for example, is not normally recognized by cariogenic organisms' transport mechanisms. In cases where transport occurs, as via a constitutive mechanism normally serving pentitols other than xylitol (cf. Scangos and Reiner [88] as a historic case with *E. coli*), the xylitol molecule is not directly involved in lactic acid production, nor is it directly involved in cariogenesis (*vide infra*). Some hexitols, such as sorbitol and *D*-mannitol can, however, be readily recognized by several strains of cariogenic mutans streptococci (Bergey's Manual of Determinative Bacteriology regards mutans streptococci as organisms that can be identified based on their positive hexitol fermentation). The absence of the reducing carbonyl group does make these hexitols less acidogenic than the corresponding aldose and ketose forms. However, sorbitol and *D*-mannitol normally support the growth of mutans streptococci and dental plaque.

(b) *The reducing power*. The sugar alcohol molecules contain an “extra” number of hydrogen atoms that can be deposited on other metabolites such as coenzymes (e.g., NADP or NAD) and other acceptors to generate chemically reduced products and intermediates of metabolism. The alditol molecules' two “extra” hydrogen atoms must thus be present in products that are formed from the alditols. The general alditol structure (CH_2_O)_n_· 2H may eventually give rise to some organic acids also in dental plaque. The regular sugars (such as glucose and fructose) have an elementary composition equalling (CH_2_O)_n_. The hexitols sorbitol and *D*-mannitol should normally yield one mole each of lactic acid, formic acid, and ethanol, while the pentitol xylitol should normally form one mole each of acetic acid, formic acid, and ethanol. For all practical purposes, the acidogenicity of dietary xylitol and erythritol, present in dental plaque, is normally insignificant.

(c) *Complexation*. Owing to the polyoxy structure of the sugar alcohol molecules they can form complex compounds with various metal cations and oxyacids. From the point of view of tooth remineralization, the complexes with Ca(II) are important. (In chemical literature, Ca(II) refers to the divalent calcium ion. In the text below, II is omitted.)

(d) *Hydrophilicity.* The presence of a large number of hydroxyl groups makes most sugar alcohols readily soluble in saliva. The most hydrophilic alditols can compete with water molecules for the hydration layer of bio-molecules (such as proteins and metal cations). Some consequences of the pronounced hydrophilicity can be seen in the strengthening of hydrophobic interactions between protein molecules (and within a protein molecule). This is in practice reflected in the protection of proteins against thermal and other denaturation or damaging processes. The protected protein configurations can include *α*-helix and *β*-structures. Related to hydrophilicity is the action of sugar alcohols as chaotropic agents under certain chemical conditions. Chaotropic agents break up organized water structures (such as the primary hydration layer of proteins and metal cations) and affect reactions that obtain their energy from the release of structured water. Another concept that will be used below, that is, the stabilization of salivary Ca phosphate systems, is a prerequisite in tooth remineralization, and results from the combined effects of complexation of Ca and displacement of water molecules.

(e) *Osmoregulator's role*. Owing to the relatively low molecular weight and the hydrophilic nature of alditols, they can function as osmoregulators in various biological systems. Perhaps the best known and most commonly exploited case concerns the use of intravenous *D*-mannitol in lowering intracranial pressure in brain surgery, in renal function studies, as a diuretic, and so forth. Sorbitol has been used as an active principal in several cathartics preparations. Erythritol (at 40 mM) exerted a significant osmoprotector effect against stress activation of corneal epithelial cells [89].

(f) *Free radical scavenging*. Because of their polyol nature, some sugar alcohols, such as *D*-mannitol, xylitol, and erythritol can act as free radical scavengers in biological and experimental systems [90].

(g) *Nucleophilicity of sugar alcohols in some hydrolytic reactions*. Several studies have shown that polyhydric compounds accelerate the hydrolysis of *β*-lactam antibiotics, *p*-nitrophenyl esters, cephalosporins, and so forth. in aqueous solutions at neutral and alkaline pH values [91, 92]. Consequently, polyhydric alcohols have shown to be catalytically active. This effect is attributed to a nucleophilic reaction mechanism affecting the molecule under attack by an alkoxide ion derived from proton ionization of one the hydroxyl groups. It is necessary to recall that several common alditols are alcohols with pK_a_ values in the range of 12 to 13. The nucleophilicity described is also displayed by other polyhydric compounds such as *D*-glucose and sucrose [92]. 

(h) *General comparison between “glucose-polyols” and “non-glucose polyols”*. The successful use of xylitol in parenteral nutrition is directly associated with the molecule's “non-glucose-polyol” nature, that is, its pentitol structure [18]. Such effects cannot be observed when sorbitol or glucose is used as a source of energy in infusion therapy [18]. This decisive difference between the xylitol and sorbitol molecules is graphically presented in Figure 2. The “glucose-polyol” nature of common dietary hexitols actually constitutes an advantage in particular nutritional uses of those hexitols such as sorbitol, *D*-mannitol, and galactitol, whereas it naturally elicits questions about the latter's stimulating effect on the growth of plaque and certain strains of mutans streptococci.

(i) *Competition between water and alditol molecules for Ca*. When alditols such as xylitol and sorbitol are introduced into the oral cavity, they compete with water molecules for the primary hydration layer of Ca. The latter may comprise 4 to 12 water molecules that surround the metal ion. The partial displacement of water molecules in the hydration layer of Ca results in the formation of a new layer consisting of alditol and water, as shown schematically in Figure 3. This interaction between alditols and Ca contribute to the stabilizing effect of polyols in salivary Ca phosphate systems [14, 18]. 

## 7. Specific Alditol Properties

In addition to all alditols displaying the above-mentioned common polyol properties, laboratory observations and computer-based examination of various structural features of the alditol series have shown that tetritol, pentitol, and hexitol is each also characterized by properties specific to individual alditols or alditol groups only. These properties have been adequately described in the relevant organic and physicochemical literature and are thus old news to chemists and biochemists. Such structural and physicochemical differences between alditols are inevitably also reflected in human and microbial metabolism. Since most tooth remineralization studies on polyols have dealt with xylitol and sorbitol, it is necessary to examine these alditols more closely. The differences between xylitol and sorbitol become evident when examining the alditol literature shown below. Some properties believed to be important in the permeability of alditols and in their relationship with water molecules are shown in Table 3.

**Figure 4:**
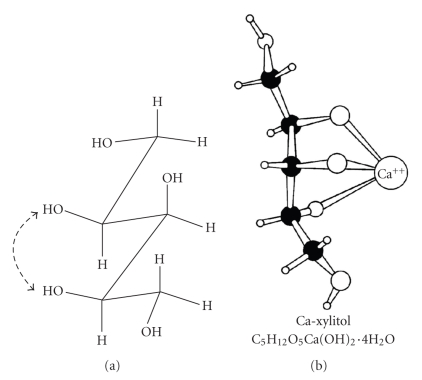
The zigzag structure of xylitol (a) and that of a xylitol-Ca complex (b) assumed to exist also in salivary environments and generally under physiologic conditions in the human body. The double-headed arrow in (a) reflects the special interaction between the oxygen atoms shown. The complex formation can facilitate the transport of Ca through membrane pores and also against weak Ca gradients. This structure may aid in the transport of Ca through the gut wall.

The scientific literature is indeed replete with descriptions on interesting differences between alditols. The very sweetening capacity of dietary alditols varies remarkably and presumes important differences between the detailed chemical structures of these homologues. Some alditols are optically inactive whereas for others a clear [*α*_D_] value has been determined (*vide supra*). The pronounced differences between the gastrointestinal tolerances of alditols in humans speak for the existence of physicochemical specificities. Extreme examples in this sense are erythritol (well tolerated) and *D*-mannitol (poorly tolerated). The very chromatographic separation of alditols (in their analytics and manufacturing) indicates the existence of important differences between individual alditols. The early studies of Mills [87] showed how the conformation of alditol exerts pronounced effects on the physical properties of the molecule, such as the retention times in gas chromatography. Further evidence is shown below:

 (1) *Historic remarks*. The epoch-making examination of Mills [87] divided alditols into several distinct series based on their stereoregular differences; the alditol series discussed show stereoregularities with short repeating units. The alditol series differ from each other, for example, with regard to the energy content of the individual homologues. Space does not allow a detailed account of the revelations that Mills and others have found between individual alditols. For example, the hydroxymethyl end-groups of galactitol can adopt their particular conformation (the so-called *A* conformation, designating the conformation round the C–C bond) more readily than the end-groups in *D-*mannitol can. This results in an important difference between galactitol and *D*-mannitol in terms of their chromatographic behaviour [87]. Analogous differences can exist also in the metabolic behaviour and clinical effects of those hexitols.

(2) *Complexation*. Although complexation can be regarded as a “common” polyol property, differences between individual alditols do exist. The hexitols, the pentitols, and the tetritols can form four, three, and two oxygen atom triangles, respectively. The exact nature of the O-triangles differs from alditol to alditol. Since these triangles can participate in complexation with Ca, complexes with different stabilities will form. The possible conformations by the interaction of alditols with partially hydrated Ca were presented by Schöllner et al. [94]. The difference between pentitols and hexitols was striking. Complex formation between polyols and divalent metal cations is a well-studied bioinorganic research area. Some remineralization-related aspects of polyol-Ca complexes have been studied [7, 14, 18, 102–104]. The proposed structure of a xylitol-Ca complex is shown in Figure 4.

(3) *Molecular parameters*. The molecular volumes, the axial lengths, and the maximum molecular radii of alditols differ significantly. The apparent molar volumes and other molecular dimensions of several alditols were measured by Kiyosawa [93], who found marked differences. The differences between the molecular weights of alditols are further reflected in numerous practical situations, such as in the permeability of alditols through membranes [93, 96]. Alditols have turned out to be valuable tools in assessing the “radius” of water-filled pores in biological systems (Table 3).

(4) *Hydration and effect on water structure*. Even pentitols differ significantly from one another, for example, regarding hydrogen bonding within the molecule. Such differences are often reflected in pronounced differences in the hygroscopicity and other properties of the pentitols [104]. Remarkable differences between the hydration properties of alditols were observed by Carlevaro et al. [105]. In line with these studies, there is a relatively strong hydration of xylitol which should be classified as positively hydrated with an extended effect on the immediate environment. The xylitol molecule exhibits a perturbational effect on water structure. Other common alditols such as erythritol, sorbitol, and* D*-mannitol exhibit negative hydration. Accordingly, xylitol can be expected to protect, for example, foodstuffs against non-enzymatic browning and ascorbic acid destruction more effectively than the above hexitols do. Related to these effects are the observed differences between alditols in their “water activity” levels [97]. This property strongly depends on the chain length and the detailed molecular configuration of the alditol. The alditol-specific effects on water structure have been known since early 1970s [95]. Polyols in general interact with water to an extent which depends on their molecular structure. For example, *D*-mannitol behaves differently from sorbitol, and glucose behaves differently from sucrose. Polyol molecules induce structure in the water molecules, surrounding them if the orientation of OH groups is such that some of the O-O spacings correspond with the O-O distance of 0.486 nm of the water lattice [95]. This effect is not a colligative property of the polyol but a property related to the OH groups.

(5) *Selective enzyme inhibition; enzyme affinity characteristics*. Xylitol inhibits more potently various microbial *D*-glucose isomerase-catalyzed reactions than the hexitols do; even the pentitols differ in this sense decisively from each other. Previous reports have tabulated representative inhibition constants (or percentage degrees of inhibition) that have demonstrated significant differences between alditols; the affinity constants of polyol dehydrogenases for their alditol substrates also differ significantly [15, 18]. Some authors have emphasized the selectivity of bacterial growth inhibition and the degree of pentose isomerase inhibition by pentitols already in the 1970s [106]. The simple alditols show distinctly different affinities for dehydrogenases involved in oxidation reactions. For example, the apparent *K_m_* values for *D*-glucitol and galactitol were 6.2 mM and 1.5 mM, respectively, in the *Rhodobacter sphaeroides* sorbitol dehydrogenase-catalyzed reactions [107]. The ribitol dehydrogenase of the same organism showed the following significantly different affinities: ribitol, 6.3 mM; xylitol, 77 mM [108]. Differences of the above magnitude, and even higher, are customarily found between alditols in enzyme kinetic studies of dehydrogenases. The pronounced differences, above, in enzyme affinities suggest that decisive differences between alditol effects can be encountered also in other biological reactions.

(6) *Kinetics of oxidation of alditols*. In addition to the above dehydrogenase-associated instances, kinetic oxidation studies of alditols offer a further point of comparison between alditols. This is a well-researched area and research findings have shown significant differences between pentitols and hexitols. For example, kinetic measurements of the rate of oxidation of xylitol and galactitol by alkaline hexacyanoferrate(III) ion showed that both oxidation reactions followed first-order kinetics with respect to hydroxide ion, but with xylitol this was true only for lower hydroxyl ion concentrations, whereas with galactitol the first-order kinetics rule applied even up to manyfold variation [109]. At lower NaOH concentrations the rate constants of oxidation of xylitol and galactitol were 0.0128 and 0.0170 mol^−2^l^2^s^−1^, respectively. The reaction progresses via an alkoxide ion, the end products being dicarboxylic acids. The difference between xylitol and galactitol was remarkable.

## 8. Alditols Are Not Tooth-Demineralizing and Calculus-Promoting Agents

The complexation between alditols and polyvalent cations may raise concern about the possible Ca-chelating (demineralizing) effect in the oral cavity. Readers unfamiliar with the true nature of complexation prevailing in the oral cavity may be misled by the terminology that is customarily used in chemistry. For example, CaSO_4_ is a relatively water-insoluble compound (at 18.75°C about 0.2 parts dissolves in 100 parts water). Yet the solubility is enhanced in the presence of, for example, sorbitol. It is important to observe, however, that the stabilizing effect of polyols on the Ca phosphate systems of the oral cavity is predominantly directed to the solubility of salivary Ca and phosphate, rendering their prolonged, dissolved, supersaturated state possible, compared with the presence of, say, sucrose, which tends to initiate instantaneous precipitation of Ca and phosphate in saliva (thus eliminating a part of those substances from remineralization). The polyols' role in saliva and plaque fluid is one of stabilization; Ca and phosphate salts are stabilized in the presence of polyols and will remain in solution even at supersaturated concentrations [14, 15, 102–104].

The polyol-Ca complexes are weak compared with those formed with common food acidulants such as adipic acid, glutaric acid, ascorbic acid, succinic acid, malic acid, tartaric acid, fumaric acid, oxalic acid, and other related carboxylic acids. Research has shown that the ability of the above food acids to chelate Ca (demineralize enamel) is directly proportional to their acidity. When present in a sorbitol candy, the amount of enamel dissolution was correlated with the potential of the acids to chelate Ca. In other words, sorbitol did not chelate enamel calcium, whereas the above acids did [110]. The same notion concerns other dietary alditols. 

Various techniques such as ultrasonic absorption, conductometry, solubility measurements, electrophoresis, and chromatography, have been used to determine that the Ca-alditol complexes are indeed relatively weak [98]. Relatively low Ca-polyol stability constants were measured for xylitol [97]. Therefore, the role normally given to alditols in the salivary environment is that of stabilization of the salivary Ca phosphate system [7, 14, 104]. This role is supposed to mimic that displayed by natural salivary peptides, such as statherin. Although the alditol-Ca complexes have thus been found to be relatively weak, they may still contribute, as Ca-carriers, to tooth remineralization and enhanced Ca absorption [18]. The consumption of xylitol has been suggested to be associated with increased Ca levels of dental plaque (Table 4). Instead of plaque hardening, (calculus formation), the extra plaque calcium is believed to enhance tooth remineralization. It is also necessary to point out that the Ca-alditol stability constants depend on temperature (stronger complexes are normally formed at lower temperatures) and that it is possible to calculate equilibrium constants for each of alditol's carbon atoms.

Although chemical analyses of dental plaque have thus shown increased Ca levels after xylitol consumption (Table 4), clinical studies on habitual xylitol users have not shown any increase in plaque mineralization; none of the clinical studies shown in Table 1 have reported on periodontal or calculus-forming problems. On the contrary, there are reports on the inflammation-dampening effects of xylitol in clinical and laboratory studies. In addition to xylitol generally reducing the growth and adhesiveness of dental plaque, xylitol formed plaque that was less inflammatory in a hamster cheek pouch microcirculation test than plaque grown in the presence of sucrose or fructose [111, 112]. Similar results were obtained in a bone culture study [113]. 5-Day-old “xylitol plaque” was less irritating to macrophages and bones than plaque grown in the presence of sucrose [114]. A study involving experimental gingivitis suggested that xylitol mouth rinses were periodontally less harmful than sucrose rinses (and equal to sodium cyclamate rinses) [100, 115]. Two clinical experiments on children indicated that the use of xylitol-containing chewable tablets and candies was associated with reduced plaque growth and gingival bleeding [116, 117]. More recently, xylitol was shown to inhibit cytokine expression by a lipopolysaccharide from *Porphyromonas gingivalis*, one of the suspected periodontopathic bacteria [118]. 

The above observations indicate that xylitol can be regarded as a periodontally safe dietary sweetener It is possible that xylitol's use can be augmented to comprise prevention of periodontal disease and gingival inflammation.

## 9. Salivary Factors Associated with Tooth Remineralization

The chemical conditions for tooth remineralization to occur can be summarized in the following five points.

1. Sufficiently high salivary (and plaque extracellular phase) pH value.
2. Sufficiently high salivary (and plaque extracellular phase) Ca level.
3. Sufficiently high salivary (and plaque extracellular phase) phosphate level.
4. Presence of natural salivary peptides that govern nucleation of hydroxyapatite crystals.
5. Presence of the required organic and inorganic matrix (i.e., the mineral-deficient enamel or dentin sites are automatically present).

Under normal conditions human saliva meets all of the above chemical prerequisites of remineralization. Consequently, saliva is normally supersaturated with regard to Ca and phosphate, and the pH value of secreted saliva normally rises spontaneously owing to the release of carbon dioxide. These conditions will automatically facilitate the precipitation of calcium phosphate. The availability of the fluoride ion within the normal, physiologic, salivary concentration range will facilitate remineralization. The phosphate component may also be partly derived from a dietary organic source, such as casein and other protein phosphates. Typical dietary phosphate sources include milk, other dairy products (cheese), and various nuts. Dietary regimens that do not elicit too-frequent acid attacks on enamel normally guarantee that the plaque extracellular phase (plaque fluid) will not become too acidic. The nucleating and crystal growth-governing salivary factors are normally present in the saliva of all normal individuals. The concentration of Ca and inorganic phosphate (P_i_) in mixed saliva, that is, 1-2 mmole/L and 2–10 mmole/L, respectively, are normally sufficient to create the required supersaturated state (of Ca phosphate) in human saliva. The overall effect of the salivary buffers gives a range of 6.2–7.4 in the saliva of most adults. These chemical conditions can be regarded as normal for tooth remineralization. Most of the buffering capacity of saliva at neutral pH values is attributed to the bicarbonate and the phosphate systems.

Saliva forms spontaneously an organic integument (the so-called acquired pellicle) on tooth surfaces. In this process extracellular enzymes secreted from plaque bacteria liberate the carbohydrate components from salivary glycoproteins (mucins), causing the residual protein structures to precipitate out of solution. The protein structures may eventually precipitate as part of the acquired pellicle film on tooth enamel. Certain salivary peptides are involved in the maintenance of the supersaturated state of Ca in saliva. Evidence has been presented on the importance of diet on the composition of the acquired pellicle; a distinct model of protein deposition on artificial hydroxyapatite discs was observed after rinses with sucrose, sorbitol, xylitol, and phosphate-buffered saline [119]. It is interesting that in the above study xylitol and sorbitol differed very significantly in Western blot tests of proteins extracted from the discs carried in the mouth for various periods of time (from 30 seconds to 20 minutes). Consequently, the alditol molecules behaved differently in the *in situ* studies of pellicle formation. The discs became saturated with protein very rapidly after each rinse, although clearly less salivary protein was adsorbed on to the discs after the sorbitol rinse than was adsorbed after xylitol rinse.

The important role salivary mucins and proteins may play in remineralization receives support from the studies of Kielbassa's group [120–122]. Mucin-based salivary substitutes (“artificial saliva”) were considered effective remineralization-inducing adjuvants that could especially benefit hyposalivation patients. Some manufacturers have marketed mucin-based saliva substitutes that also contain xylitol.

## 10. Ca-Binding in Dental Plaque

As shown above, xylitol consumption has been found to be associated with an increase of plaque Ca levels (Table 4). The extra Ca (compared with control situations) present in dental plaque in its entirety and in plaque extracellular fluid in particular may participate in the remineralization of mineral-deficient enamel sites. Approximately half of the concentration of plaque Ca may be ionized. It is not yet known how much of the Ca present in “xylitol plaque” is in an ionized form. It is known, however, that polyols can facilitate the solubilization of the insoluble portion of plaque Ca. It is possible that the increased Ca inhibits demineralization through a common ion effect, and may additionally facilitate remineralization during periods of high pH values.

It is necessary to recall that Ca-binding by various Gram-positive plaque bacterial strains is a common phenomenon [123, 124]. Ca-binding by various surface components of oral bacteria may indeed exert significant effects on remineralization/demineralization processes. Various oral-care products such as mouth rinses and chewing gum have been used to increase the plaque levels of a combination of casein phosphopeptides and amorphous Ca phosphate (CPP-ACP), and to facilitate remineralization of enamel [125, 126]. Incorporation of CCP-ACP into plaque naturally also increases the plaque levels of Ca and P_i_. In one mouth rinse study, the plaque Ca and P_i_ levels increased by 118% and 57%, respectively [126]. These authors strongly believe that the extra plaque Ca can significantly contribute to tooth remineralization.

The xylitol-associated increase of plaque Ca levels (Table 4) is most likely a general polyol-associated reaction; xylitol and sorbitol may not differ remarkably in this sense. The extra Ca is believed to contribute to tooth mineralization during a polyol regimen. Sorbitol, however, normally supports the growth of dental plaque and mutans streptococci.

Enamel permeability naturally plays a role in rendering Ca available for remineralization. The permeability of common salivary and dietary inorganic ions (such as Ca and P_i_) has been discussed in several contexts, but less attention may have been paid to the permeability of common dietary carbohydrates. In this sense it is necessary recall that according to some permeability studies twice as much xylitol goes through the enamel as sucrose [127].

Polyols generally stabilize the calcium phosphate solutions in saliva [97]. This phenomenon results from the formation of complexes between Ca and the polyol. Although ketoses, aldoses, disaccharides, and other carbohydrates also form complexes, those formed between xylitol and Ca are of particular interest owing to the general nonacidogenicity of xylitol in the human oral cavity. The stabilizing effect of xylitol on the salivary Ca-phosphate system can easily be demonstrated by letting acellular (Millipore-treated) whole-mouth saliva stand at room temperature in the presence or either sucrose or xylitol. Sucrose allows almost instantaneous precipitation of Ca-proteinates, whereas the turbidity formation is significantly delayed in the presence of xylitol [102, 103]. In other words, xylitol mimics some of the salivary peptides (such as statherin) which control crystal formation. It has been assumed that sucrose effectively eliminates from the solution the “extra” calcium and phosphate ions that in the presence of xylitol can maintain their supersaturated, natural concentration level, a prerequisite of remineralization. In one study, saliva precipitates contained a crystalline phase that had the structure of apatite [104]. Xylitol maintains a higher pH value in saliva and plaque fluid and simultaneously maintains a supersaturated Ca-level in saliva. The combined effect can manifest as remineralization that is governed in the same way as salivary peptides.

## 11. Formation of Basic Equivalents

**Figure 5:**
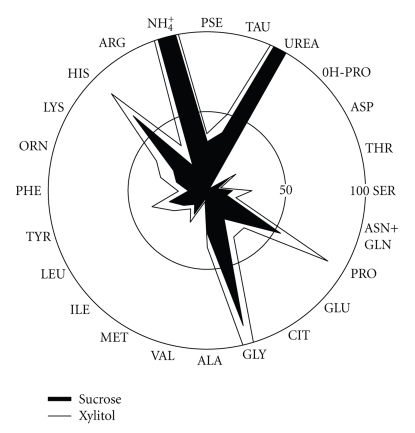
An important sialochemical effect of xylitol diet. Increase of the free amino acid content (in *μ*mol/L, thin line) of whole-mouth saliva after long-term consumption of a xylitol diet, shown in a polar co-ordination diagram [13]. The analysis was carried out on pooled saliva of subjects who had consumed the xylitol diet for 12 to 16.5 months (average consumption level of xylitol: about 65 g/day). The solid black area shows the free amino acid levels in saliva of subjects who consumed a regular sugar-containing diet. The high levels of ammonia and most amino acids (which can in turn serve as sources of further ammonia production) speak for reduced plaque acidity and increased nitrogen metabolism in dental plaque from which a large part of the free amino acid pool of whole-mouth saliva is derived. Non-standard abbreviations: CIT = citrulline; TAU = taurine; PSE = phosphoserine. Reproduced with permission [14].

Because the rate of tooth remineralization generally increases already in slightly alkaline conditions, it is pertinent to recall the increase in dental plaque and whole saliva of various basic substances during xylitol regimen. The first clue of this type biochemical effects was obtained in the Turku Sugar Studies [13] which showed that the consumption of a xylitol diet was associated with a general increase of nitrogen- and protein-containing substances in saliva. Among such substances were amino acids (which in turn can generate ammonia), and the ammonium ion itself [13, 14]. The pie chart in Figure 5 demonstrates the general increase in ninhydrin-positive substances (mostly amino acids) present in the whole-mouth saliva of subjects who habitually consumed a xylitol diet for 12 to 16.5 months. Other studies have also shown that the stimulation of saliva with xylitol products increases the ammonia [116] and the bicarbonate [13] content of whole-mouth saliva.

## 12. Description of Individual Remineralization Studies

The list below summarizes in chronological order 27 separate remineralization-associated clinical, basic science, and animal studies on xylitol and other sugar alcohols (two studies, #19 and #26, investigated enamel erosion). The list begins with the Turku Sugar Studies [13] whose authors used the phrase “remineralizing and therapeutic effect of xylitol” in their first clinical reports on xylitol-associated limitation of dental caries [128].

**(1) Remineralization of Caries Lesions During a Two-Year Xylitol Feeding Trial and in a One-Year Chewing Gum Study**

The final report of the two-year Turku feeding trial showed that the consumption of a xylitol diet was associated with the reversal of the caries process [65]. Because essentially similar observations were also made in a simultaneously conducted one-year chewing gum study [129], the authors of the trials were led to conclude that the usage of xylitol was indeed associated with remineralization of caries lesions [13]. Habitual use of xylitol gum resulted in a negative development of the DMFS index scores, an indication of remineralization. Subsequent planimetric evaluations of the lesion size reductions after xylitol usage in both studies supported the original conclusions made [130, 131]. An interesting, partly accidental outcome of these trials was that while the daily consumption level of xylitol in the two-year feeding trial was estimated to be 67 g per subject, the intake of xylitol in the one-year gum study amounted to one tenth of the above value, that is, 6.7 g. Partly owing to these consumption levels, the Finnish health authorities have recommended the use of 6 to 7 g xylitol daily in the prevention of caries. This recommendation has been widely followed in the instructions issued by national dental associations in several Asian countries [30].

**(2) Effect of Xylitol-Supplemented Diet on the Regression of Fissure Caries in Rats**

Dentinal molar fissure caries in rats, produced by initial exposure to dietary sucrose, were significantly reversed by subsequent exposure to a xylitol-supplemented (3% and 6%; about 0.2 M and 0.4 M, resp.) starch diet. Continued exposure to a xylitol-starch diet produced a successive regression of caries rates, which the authors interpreted as remineralization and as a therapeutic effect [7, 62].

**(3) Remineralization of Artificial Caries-Like Lesions by a Xylitol-Containing Mouth Rinse**

One of the mouth rinses tested by Featherstone et al. [132] contained 2.5% (0.164 M) xylitol “to mask the mineral taste and produce a palatable rinse”. Other ingredients included 0.6 mM NaF, and K, Ca, Sr, and Zn salts. Human enamel slabs with demineralized lesions were embedded in intra-oral appliances. Complete rehardening of the inner 40–50 *μ*m and twofold rehardening of the remaining body of the lesion occurred in 160-*μ*m deep lesions with a 1-min mouth rinse on each of 14 consecutive days. Saliva re-hardened the inner 20 *μ*m only. The authors did not pay attention to the possible role of xylitol as a remineralization-contributing factor.

**(4) Remineralizing Properties of Xylitol in Combination with Sucrose in Rats**

Rats, inoculated with *Streptococcus mutans*, were fed a diet containing 20% sucrose, 5% glucose, and 5% xylitol (SX diet), or solely a sucrose-glucose diet (S). (In molar terms, the above concentrations are about 0.58 M, 0.28 M, and 0.33 M, resp.) SX induced significantly fewer fissure lesions than S. Initial lesions induced by S were significantly reduced (remineralized) by subsequent exposure to SX. Change from SX to S resulted in substantial caries progression [64].

**(5) Rehardening Properties of Xylitol-CMC-Containing Saliva Substitute**

The rehardening of artificially softened human enamel by nine different saliva substitutes was investigated by micro-hardness measurements [133]. All saliva substitutes exhibited a rehardening potential. The largest reduction of the rehardening potential was observed after addition of high concentrations of carboxymethylcellulose (CMC) or mucins and the addition of sorbitol (3% in the substitute; 0.165 M). The best rehardening properties were observed for low-viscous mucin- or CMC-containing saliva substitutes with xylitol (2% or 0.13 M; Saliva Orthana, Copenhagen, Denmark). The difference between xylitol and sorbitol was significant (*P* < .01).

**(6) Influence of Xylitol and Sucrose on Enamel Demineralization In Vivo**

Fissure-like plaque retention grooves were created in human enamel blocks and demineralized. The blocks were mounted in prostheses of 11 subjects who used a 2.5% (0.164 M) xylitol, 2.5% (0.073 M) sucrose, or a water solution in a randomized cross-over design. During a period of 16 days, the subjects submerged the prosthesis twice a day in the test solutions for 5 minutes. Mineral loss and lesion depth were measured before and after the experiment by means of quantitative microradiography and polarized light microscopy. At the surface enamel, a significant reduction of enamel demineralization was found after the xylitol treatment. The lesion depth at the surface enamel increased 17 *μ*m after sucrose and 7 *μ*m after xylitol (*P* < .05) [134]. Similar enamel blocks were also treated for 28 days with 35% (2.3 M) xylitol toothpaste [135]. The results were in line with the above study.

**(7) Effect of Xylitol, Lactitol, and Other Sweeteners on Tooth Demineralization**

Mixed cultures of dental plaque organisms were incubated for 24 hours in media containing different sweeteners. The attack of acids so generated was measured by Ca and phosphorus analyses. Demineralization was most severe with glucose and sucrose. Lactitol and xylitol showed extremely low enamel demineralization figures [136].

**(8) Effect of Fluoride-Polyol Gum on Remineralization**

Maxillary acrylic appliances carrying carious enamel sections were worn by subjects who used Fluogum (0.113 mg F/stick) that also contains xylitol and sorbitol. After three days of chewing 15 sticks there was a significant reduction in both lesion depth and in the size of the body of the lesions by an average of 5% (*P* < .05) [137]. It is possible that both polyols contributed to remineralization.

**(9) Combined Effect of Xylitol and Fluoride on Enamel Demineralization In Vitro**

Bovine enamel was exposed to a buffered lactic acid solution at pH 4.5 at +37°C. The selected enamel windows were treated with 2.63 M xylitol, 0.3 mM NaF, and their combination. Control enamel was not treated. The lesion depth was assayed by means of transversal microradiography. Xylitol and NaF had comparable effects on lesion reduction. The effects of F^−^ and xylitol were additive [138].

**(10) Effect of Sorbitol Chewing Gum on Enamel Lesion Remineralization**

Subjects wore *in situ* appliances on which were mounted enamel sections containing artificial caries lesions [139]. Sorbitol-containing gum (the U.S. brand Orbit Extra) was chewed by volunteers for 20 minutes 5 times a day over a period of 7 weeks according to a cross-over design. Microradiography showed that gum chewing caused 18.2% remineralization compared with the control's 12.1% remineralization (*P* = .07). However, a regular sucrose gum, used in an identical protocol, also caused significant remineralization (18.3% versus 10.8% in the control). The latter observation and the long chewing time used in this study suggest that any possible, specific polyol effect was possibly masked by a common “salivary effect”. Hence, no proof was obtained for a specific remineralization-enhancing effect of sorbitol itself.

**(11) Effects of Sucralose, Xylitol, and Sorbitol on Remineralization of Rat Caries**

Rats infected with *Streptococcus sobrinus* were first given drinking water containing 10% sucrose. A group of animals were thereafter fed either the same sucrose water, or received their nutrition by gavage and drank water containing 0.03% sucralose, 10% xylitol, or 17% sorbitol (added at a sweetness equivalent to 10% sucrose). The authors [140] reported that removal of the cariogenic challenge (sucrose) allowed remineralization to occur and that no sweetening agent was superior to another in this respect. The following details of the study deserve attention. (1) The intervention lasted only three weeks. (2) The rats were mono-infected with one bacterial species. (3) The authors' attempt to create almost isosweet drinking water for the animals resulted in significant differences in the chemical concentrations of the sweeteners used: sucrose 0.292 M, sorbitol 0.934 M, xylitol 0.658 M, and sucralose 0.876 mM. These characteristics of the study make it impossible to draw conclusions on the relative ability of the named sweeteners to facilitate remineralization or to reverse the process of “natural” caries.

**(12) Effects of Sorbitol and Sorbitol/Xylitol Chewing Gums on Human Enamel Remineralization**

Intraoral remineralization of experimental caries-like lesions in human enamel was studied by Manning et al. [141]. Polarized light microscopy and quantitative microradiography showed that 20-min chewing of a sorbitol gum and a 3 : 1 sorbitol/xylitol gum 5 times per day over a period of 21 days promoted remineralization to a similar extent. The following features of the study deserve attention: (1) The long chewing time used may have abolished any possible, specific polyol effects. (2) Both chewing gums contained sorbitol as the only or as the clearly predominating polyol sweetener. Hence, it may be difficult to compare the remineralization-enhancing effect of xylitol and sorbitol in this study.

**(13) Xylitol-Induced Changes of Enamel Microhardness after Consumption of Xylitol Candy**

Slabs of bovine enamel were inserted in cavities of children with rampant caries. The negative control subjects did not receive sweets whereas the treated subjects received 20 g xylitol daily in the form of candies. Predemineralized and non-demineralized enamel showed pronounced, statistically significant rehardening at exposure to xylitol (*P* < .001). Microradiography confirmed these findings [142].

**(14) Effect of Xylitol and Sorbitol in Chewing-Gums on Mineral Loss of Enamel**

Human subjects with >3 × 10^5^ mutans streptococci per ml of saliva participated in a cross-over study involving the use of four different chewing-gums containing: (1) 70% xylitol, (2) 35% xylitol + 35% sorbitol, (3) 17.5% xylitol + 52.5% sorbitol, and (4) 70% sorbitol [143]. The subjects used 12 pieces of each gum (1.6 g) per day for 25 days. The wash-out periods lasted about 10 weeks. The subjects wore a removable palatinal plate containing two demineralized human enamel samples. The authors reported that the lesion depth and the mineral loss values, assessed microradiographically, did not differ significantly between groups. Increased concentration of xylitol in the gum resulted in a lower number of mutans streptococci in saliva and dental plaque. However, the pH drop in plaque measured in vivo after a 1-minute mouthrinse with a 10% sorbitol solution was least pronounced after the 70% xylitol gum and most pronounced after the 70% sorbitol gum period (*P* < .01). It is possible that the cross-over study design, the shorter wash-out periods, and shortness of the treatment affected the microradiographical data [144].

**(15) Stabilization (Remineralization) of Rampant Caries by the Use of Polyol Chewing Gums**

The results from two cohort studies on arrest and non-progression of dentin caries after long-term usage of xylitol- and sorbitol-containing chewing gums by young subjects are summarized [145, 146]. The original research papers [61, 72] suggested that 40- and 24-month consumption of xylitol-containing chewing gum was superior to sorbitol-containing gum in the arrest of dentin and enamel caries. A re-examination 5 years after the 24-month study lent further support to this finding [83].

**(16) Remineralization of Dentin Caries Lesions of Primary Teeth by Means of Physical, Chemical, and Histologic Procedures**

The Stann Creek study in Belize showed that habitual use of xylitol gum was associated with arrest of dental caries in young subjects [72]. After a 20–22-month intervention (when the children were 8 years old), a total of 23 primary teeth with extensive dentin caries lesions, whose surface in clinical examination was found to be totally remineralized, could be removed because the teeth were near their physiologic exfoliation time. The majority of the specimens had been remineralized from the surface by a non-cellular-mediated process. The topmost 20-*μ*m layer of the lesions exhibited the highest Ca : P ratio. The rehardened surface layer (normally <0.1 mm in thickness) was significantly (*P* < .001) harder than sound dentin and nearly as hard as sound enamel. The extracellular remineralization was most likely mediated by odontoblasts [147]. The bioinorganic and physicochemical mechanisms behind these effects are outlined [14, 15, 102, 104].

**(17) Timing of First Restorations in Relation to a Preventive Xylitol Trial**

Re-examination 3 and 5 years after a 2-to 3-year xylitol gum program suggested that xylitol had “permanently prevented caries” [80]. An independent retrospective analysis showed that the need for restorations was significantly postponed in xylitol-receiving subjects [81].

**(18) Rehardening of Enamel Caries Lesions after a 16-Month Xylitol Gum Program That was Preceded by a 40-month Sucrose Gum Program**

The 40-month Belize chewing gum program showed that the use of sucrose gum by subjects with high dietary sugar consumption was associated with high caries activity. After the termination of the 40-month trial, subjects of the sucrose gum group were recruited to an intense xylitol gum program (xylitol consumption level: 14 g per day and subject) for 16 months. The intensified xylitol gum usage was associated with a significant reduction in the mean DMFS score (*P* = .0013). The reduction of the score most likely resulted from the change of the *D* component of the index and possibly reflected rehardening of some caries lesions to a non-progressive carious state [82].

**(19) Influence of Xylitol and NaF on Dental Erosion In Vitro**

Sectioned bovine incisors were treated with orange juice only or with juice containing 25% (1.64 M) xylitol and/or 0.5 ppm F. The samples were immersed in the test solutions six times daily for 5 min on each occasion for 24 days (total exposure: 12 hours). Mineral loss measurements showed that xylitol and F had an additive effect on the reduction of dental erosion by orange juice in vitro [148]. Using a similar study design, the same authors concluded that “tolerable levels of xylitol alone may not show significant caries inhibiting and remineralizing effect, but may act as a caries inhibitor additively with fluoride” [149].

**(20) Japanese Remineralization Experiments with Xylitol and Ca Hydrogenphosphate**

Yanagisawa and his co-workers [150, 151] have provided crystallographic evidence on the restorative process of xylitol-associated remineralization. In their studies human enamel specimens were demineralized in acetate buffer at pH 4.0 and subsequently immersed at 37°C for two weeks in a remineralizing solution containing Ca, phosphate, F, and 20% xylitol (control solutions did not contain xylitol). Contact microradiography showed that, in the absence of xylitol, remineralization was mainly observed in the surface layers of the samples. In the presence of xylitol, somewhat less remineralization was observed in the outermost surface layers (up to 10 *μ*m), whereas the middle and deeper layers exhibited pronounced remineralization. Multipurpose image processor studies confirmed this observation. It was concluded that xylitol acted as a carrier of Ca, maintaining a constant Ca level by introducing more mineral from the surface layers into the middle and deeper zones. Eventually, in the presence of xylitol, remineralization occurred over the entire demineralized zone [152, 153]. Further studies demonstrated that a combination of xylitol and Ca hydrogenphosphate was more effective than this same Ca salt supplemented with erythritol, sorbitol, maltitol, or palatinit [154]. When funoran (a sulphated polysaccharide of the seaweed *Gloipeltis furcata*) was added to the xylitol-Ca salt mixture, an increase in the extent of remineralization was observed [155]. Another study showed that the use of a chewing gum containing xylitol, Ca hydrogenphosphate, and funoran was more effective than a similar gum containing maltitol [156]. The inhibitory effect of funoran on the adherence and colonization of oral bacteria was further clarified by Saeki [157–159]. Recently, Japanese and Thai researchers teamed up for studies which supported the above concept, that is, the remineralization-associated influence of xylitol chewing gum containing funoran and calcium hydrogenphosphate [160].

**(21) Remineralization of Enamel Lesions by Various Forms of Ca in Mouth Rinse or Chewing Gum**

Casein phosphopeptide and amorphous Ca phosphate nanocomplexes (CPP-ACP) have been intensively tested by Reynolds's research team [126, 161]. The CPP-ACP complex was tested in two different Lotte (Tokyo, Japan) xylitol gum formulas (one containing xylitol and CaCO_3_, the other xylitol, CaHPO_4_/CaCO_3_, and funoran). Several CPP-ACP mouth rinse preparations were also tested. The xylitol-CPP-ACP and the sorbitol-CPP-ACP combinations prevented demineralization and promoted remineralization [126]. Xylitol and sorbitol gums did not differ [161]. These studies have employed a cross-over design with wash-out periods of only one week or somewhat longer between interventions. Owing to the fact that xylitol can exert long-term effects on dental caries [144], it is impossible to draw firm conclusions from these studies as to the relative ability of polyols to enhance remineralization.

**(22) Effect of Polyol-Containing Saliva Substitutes on Demineralized Dentin Specimens**

Bovine dentin specimens were demineralized for 14 days at pH 5.5 and subsequently treated for 14 days with mucin- and CMC-based saliva substitutes that also contained 2% xylitol or sorbitol. Loss of mineral was assayed by means of transversal microradiography. For the dentin specimens, significant (*P* < .05) differences were observed between xylitol and sorbitol, the xylitol-containing saliva substitutes displaying lower mineral loss values. For enamel specimens, no significant differences were observed [162].

**(23) Effect of Xylitol + Ca Lactate on Remineralization**

Artificial caries-like lesions were created in human enamel slabs which were subsequently worn by volunteers who used, in a three-leg study, over a period of two weeks, chewing gum containing either xylitol or xylitol + Ca lactate, or did not receive any gum [163]. X-ray spectrometry showed that the xylitol-Ca combination remineralized the lesions more effectively than the xylitol and the no-gum periods.

**(24) Caries Prevention with Xylitol-Containing Hard Candies among Disabled Pupils**

A field study of 18 months' intervention was carried out in physically disabled school children, who received about 2.3 g xylitol in the form of hard candies in three daily episodes. The control subjects did not receive xylitol candies. The authors concluded that xylitol had “a strong and a clear remineralizing effect on caries” [57].

**(25) Effect of Xylitol + F Toothpaste on the Remineralization of Enamel In Vitro**

Human enamel specimens, demineralized at pH 4.5, were treated twice a day for 14 days in a silica-based toothpaste slurry containing 500 ppm F, 500 ppm F + 5% xylitol, or no added F and xylitol. Quantitative light-induced fluorescence studies showed that the combination of xylitol and F toothpaste was superior to the other treatments in occasioning remineralization of demineralized enamel [164].

**(26) Effect of Xylitol and Fluoride on Enamel Erosion In Vitro**

Since enamel erosion and tooth demineralization are chemically partly similar processes, one human enamel erosion study will be mentioned below. Human third molars were immersed for one min or five min in various test solutions four times a day for 14 days. Mineral loss was determined from lesion depth and surface hardness. Addition of xylitol, NaF, or a xylitol/NaF combination to an acidic drink (orange juice) reduced (but did not prevent) enamel erosion [165].

**(27) Effect of Isomalt on Enamel De- and Remineralization**

In an in vitro study, subsurface bovine enamel lesions were subjected to 3-week pH-cycling involving 5-minute rinses with 10% isomalt (*α*-*D*-glucopyranosyl-1,6-sorbitol) solutions daily and 10% isomalt additions to re- or demineralizing solutions [166]. A 0.2 ppm fluoride “background” was used during the remineralization phase. In an *in situ* study, subsurface lesions were exposed for 2 months in vivo and brushed 3 times a day with a tooth paste containing 10% isomalt. In the in vitro study, 5-min rinses resulted in slightly increased remineralization, while continuous presence of 10% isomalt (in re- or demineralizing solutions) inhibited de- and/or demineralization. The *in situ* test confirmed enhancement of remineralization. It is possible that isomalt stabilizes the calcium phosphate system present in enamel surface.

## 13. Erythritol

The noncariogenicity of erythritol has been investigated in rats [167] and, based on the presence of various risk factors of caries (such as mutans streptococci and dental plaque), erythritol has been regarded as noncariogenic in humans [168–170]. All studies strongly support the idea of erythritol as a caries-reducing dietary polyol. Especially the way erythritol inhibited the growth of certain mutans streptococci isolates is interesting [170]; the results indicate that the mechanism of growth inhibition differs from that caused by xylitol. Partly based on such experiments, it is tempting to postulate that certain combinations of erythritol and xylitol will turn out to exert promising caries-limiting effects in humans. The combined effects may exceed or at least equal the separate effects of these polyols. 

Also studies on erythritol have re-emphasized the inevitability of the existence of important differences between individual alditols; the alditols cannot be regarded as an entity with exactly identical molecular parameters and similar biological effects. Such a contention would be incongruent with accepted physical and chemical laws. It would be space-consuming to list all separate studies that have demonstrated the existence of selective alditol effects. In the case of erythritol, however, the following examples may serve a purpose: Sugar alcohols, especially erythritol, enhanced the fungicidal effect of benzethonium chloride toward in vitro candidal biofilms; the difference with xylitol and *D*-glucitol (sorbitol) was significant [171]. De Cock and Bechert [90] emphasized the free-radical-quenching effect of erythritol, a property which may play a role in the “functionality” of erythritol-containing food products.

Several erythritol caries trials have been initiated in various parts of the world. Although there is still no valid clinical proof, the known molecular parameters of simple dietary alditols suggest that their caries-limiting efficacy may follow the simple homologous alditol series, will depend on the number of hydroxyl groups present in the alditol molecule, and will decrease as follows: erythritol ≥ xylitol > sorbitol. Combinations of erythritol and xylitol may have an edge over either of the alditols used separately: their microbiologic mechanism of action in caries prevention seems to differ and they are believed to exert a concerted and additive effect. However, no long-term human caries trial on erythritol has been completed. Hence, the possible difference between erythritol and xylitol in terms of caries-limiting ability will hopefully be elucidated in the near future. It will be essential to carry out trials where all of the above polyols (one tetritol, one pentitol, and one hexitol) will be simultaneously tested. The excellent gastrointestinal tolerability of peroral erythritol even in infants may promote its combination with xylitol in caries-limiting strategies.

## 14. Addendum

The following research papers and statements have been published after the above text was completed. 

The effect of xylitol chewing gum on the acquisition pattern of 39 bacterial species (including mutans streptococci) was investigated in infants [172]. Mothers used xylitol or sorbitol gum (4.2 g/day) or no gum; gums were used 3 times a day for 9 months. The authors concluded that maternal use of xylitol gum did not result in statistically significant differences in the microbial plaque composition of 9- to 14-month-old infants. These results partly contradict those obtained in Finnish and Swedish mother-child studies (Table 1) where the counts of mutans streptococci decreased in children whose mothers had used xylitol. Perhaps the treatment period should have been extended beyond 14 months since the “window of infectivity” for mutans streptococci does remain open longer than 14 months. It is also possible that the polyol level in the tested gums was too low.

The following study completes caries trial information of Table 1: Pediatric topical oral xylitol syrup was administered in a group of 94 children aged 9 to 15 months for about 10.5 months. Parents administered syrup twice a day (2 xylitol 4 g doses and 1 sorbitol dose) (Xyl-2x group) or three times per day (3 xylitol 2.67 g doses) (Xyl-3x group) versus a control syrup (1 xylitol 2.67 g dose and 2 sorbitol doses). The data showed that oral xylitol syrup administered topically 2 or 3 times daily at a total daily dose of 8 g was effective in preventing early childhood caries [173]. These findings seem to be in congruence with previous field experience (Table 1) and also with the theory of the pharmacologic mode of action of xylitol in caries limitation [18].

The concept of the formation of oral biofilm has emerged during the last decades to facilitate the understanding of physiological oral processes. A recent study suggested that xylitol not only effectively inhibits acid production of cariogenic bacteria, but also prevents the formation of a multi-species bio-film [174]. In many instances oral bio-film is tantamount to dental plaque.

It is noteworthy that recently published anonymous dental journal editorials have emphasized the role of xylitol in caries prevention [175–177]. In an article in The Journal of American Dental Association [175] the potential role of xylitol-containing oral syrup in the prevention of childhood caries was discussed. The European Union also finally cleared xylitol for certain anti-caries claims [176]. The American Academy of Pediatric Dentistry's Council on Clinical Affairs issued its policy on the use of xylitol in caries prevention [177]. The resolutions and endorsements contained in the above texts are reflected in recent conclusions of leading experts on the use of polyol gums to prevent dental caries in general [178] and especially in early childhood [179, 180].

Finally, a recent research paper has shown that xylitol can indeed modify dental plaque, resulting in marked reduction in plaque acidogenicity (which was not detected using *D*-glucitol) [181], a finding that confirms similar results obtained almost 20 years earlier [182, 183]. Previously observed morphologic effects of xylitol on *S. mutans* [184] received partial support from a recent study [185] which showed that chewing xylitol gum over one year may negatively affect the synthesis of extracellular polysaccharides by *S. mutans* (because the adherence of colonies in xylitol-receiving subjects decreased). Related to these findings is the observation that both erythritol and xylitol can decrease polysaccharide-mediated cell adherence that contributes to plaque accumulation [186]. Since cooked starch can be regarded as potentially cariogenic, it is noteworthy that xylitol, along with maltotriitol (“gluco-gluco-sorbitol”; derived from maltotriose) and acarbose (a pseudotetrasaccharide containing an unsaturated cyclitol moiety; an *α*-glucosidase inhibitor) decreased the production of acid from starch by *S. mutans* and *S. sobrinus* [187]. 

## 15. Conclusions

Dental caries is a multi-factorial, diet-associated infectious disease that initiates as minor calcium-deficient lesions in tooth enamel. The repair (remineralization) of minor enamel defects is a normal physiological process that is well known to clinicians and researchers in dentistry and oral biology. This process can be facilitated by various dietary and oral hygiene procedures. The remineralization (rehardening) process may also concern dentin caries lesions. Consequently, the disease (dental caries) is reversible, if detected and treated sufficiently early. The scientific and clinical information available today indicates that habitual use of xylitol, a sugar alcohol of the pentitol type, can be associated with significant reduction in the incidence of dental caries and with remineralization of both enamel and dentin caries lesions. Suitable xylitol-containing “vehicles” include chewing gum, hard candies, gum arabic-based saliva stimulants, and dentifrice. Mouth rinses (“artificial saliva”) and fluoride gels aimed at hyposalivation and at special dental caries patients, respectively, also serve as clinically effective oral health adjuvants. Other dietary polyols that can remarkably lower the incidence of caries include erythritol which is a tetritol-type alditol. Based on the molecular parameters of simple dietary alditols, it is conceivable to predict that their efficacy in caries reduction will follow the homologous series, that is, that the number of hydroxyl groups present in the alditol molecule will determine the efficacy as follows: erythritol ≥ xylitol > sorbitol (the possible difference between erythritol and xylitol has not yet been adequately established). Most caries-related information available today is focused on the effects of xylitol and sorbitol. The present review examines the physical, bioinorganic, and biological chemistry of alditols from points of view that are believed to play a role in oral biology and caries prevention. The review also provides an account of tooth remineralization studies carried out with xylitol and sorbitol, as well as reports on recent caries-associated findings on erythritol.

## Figures and Tables

**Table 1:** Summary of human caries studies on xylitol that in part have constituted the justifications for public endorsements of xylitol. The percent-reductions are in comparison with a control group that received a normal diet, fluoride treatment, or sucrose products. Nondietary (dentifrice) studies and programs on multiple preventive measures that included the use of xylitol are also shown. CH = Chlorhexidine.

Study location	Product(s) tested	Duration (years)	Dose (g/day)	Caries reduction (%). Comments. (References).
Finland	Full diet	2	67	>85. Compared with sucrose diet [13, 65]. Mostly adults.

Finland	Chewing gum	1	6.7	>82. Compared with sugar gum. 1/10 of the above dosage [13, 65]. Young adults.

Soviet Union	Candies	2	30	Up to 73. Compared with sucrose candies [66].

French Polynesia	Chewing gum	3	About 20	58–62. Compared with normal diet [67].

Hungary	Gum, candies, dentifrice	2-3	14–20	37–45. Compared with fluoride [68, 69].

Canada	Chewing gum	1-2	1.0–3.9	52. [70].

Finland	Chewing gum	2	7–10	30–57. All subjects (no-gum as control)^(a)^. [71].

Finland	Chewing gum	3	7–10	59–84. High-risk subjects^(a)^. [71].

Costa Rica	Dentifrice + NaF	3	Twice/day	Up to 12.3. 10% xylitol in the product. [44].

Costa Rica	Dentifrice + Na_2_FPO_3_	3	Twice/day	Up to 10. 10% xylitol in the product. [45].

Belize	Chewing gum	3.3	<10.7	Up to 73. Permanent teeth^(b)^. [61].

Belize	Chewing gum	2	<10.7	Up to 63. Deciduous teeth^(c)^. [72].

USA	Gum, pastilles	1.8	8.5	80. Supragingival root surface caries. Elderly subjects [73].

Estonia	Gum, pastilles	2-3	5	50–60. Used on school days^(d)^. [51]. Pastille as effective as gum.

Finland	Chewing gum (by mothers)	ca. 1.75	6	70 (in children). NaF and CH as control [74].

Lithuania	Chewing gum	3	2.95	21–36. [35]. Rectification of initial results [36]^(e)^.

Sweden	Chewing gum (by mothers)	1	2	“Significant” or 40% (in children). [75, 76]^(f)^.

Kuwait	Hard caramels	1.5	2.3	50. Läkerol-type hard candies were used [57]^(g)^.

Finland	“Slow-release pacifier”	1	159 mg	No new dentinal lesions in infants [46]. A mixture of xylitol, sorbitol, and NaF was tested. The pacifier features a pocket for the sweetened tablet.

Finland	Multiple measures	About 3.4	4.6	Counselling and the use of fluoride- and xylitol products reduced caries (*P* < .001) compared with basic prevention [77]^(i)^.

^(a)^Long-term effects (after up to 5-year use) have been reported [78–80]. An independent analysis showed that the total number of new restored surfaces was 4.0 per child in the xylitol group and 9.3 in the controls during the decade after the onset of the trial. Participation in the xylitol gum trial thus resulted in significant reduction in the number of first restorations and hence in costs during the subsequent decade [81].

^(b)^16-month use of xylitol gum following the 3.3-year use of sucrose gum reduced caries significantly [82]. “<10.7” indicates the maximum calculated, supervised use (at school) per day and subject.

^(c)^Two-year use of xylitol gum remarkably protected erupting permanent teeth against caries, that is, long-term effects were involved [83].

^(d)^Saliva stimulants were given only on school days (about 200 per school year). Gums were as effective as pastilles (hard candies of the “Läkerol-type”).

^(e)^The original authors failed to recognize that, in their study, xylitol gum was the only gum that lowered the DMFS increment compared with the no-gum group after 3 years. “To still observe a significant caries-lowering effect of xylitol with such a small dosage is quite remarkable”. The faulty conclusions were rectified by Hayes [36].

^(f)^In one literature source, the authors reported an 80% reduction between “test and control”. Also, when the children were 18 months old, the authors reported that “maternal consumption of xylitol- and CH/xylitol-containing chewing gums significantly reduced the mother-child transmission of salivary mutans streptococci”. This study actually compared a gum with high xylitol content with gums with lower xylitol content, supplemented with either CH or NaF.

^(g)^Xylitol hard candies were given only on school days (one piece of candy at a time, three times a day).

^(h)^The pacifier features a pocket from which the saliva stimulants dissolve.

^(i)^The Läkerol Dents brand (Leaf). The products were given to the subjects with instructions “to be used according to directions” (i.e., two pieces of candy three times a day). The calculated maximum consumption level of xylitol was about 4.6 g/day.

**Table 2:** PubMed literature search for “Tooth Remineralization”.

Years	Number of references	References per year
1966–1975	1	0.1
1976–1984	10	1.3
1985–1990	180	36.0
1991–1995	156	39.0
1996–1999	136	45.3
2000–2004	222	44.4
2005–2008	228	57.0

**Table 3:** Physicochemical properties of alditols at 25°*C*.

Alditol	Molecular weight	Maxium van der Waals radius (Å)^(a)^	Partial molar volume (cm^3^ mol^−1^)^(b)^	Permeability (m s^−1^)^(c)^	“Water activity” constant *K* ^(d)^
Glycerol	92.1	2.8	70.84	1.49 ± 0.40 × 10^−10^	1.16
Erythritol	122.1	3.1–3.2	86.83	4.92 ± 0.27 × 10^−10^	1.34
Xylitol	152.1	3.2–3.3	102.12	9.9 ± 3.4 × 10^−11^	1.66
*D*-Arabitol	152.1	3.2			1.41
*L*-Arabitol	152.1	3.2			1.21
Ribitol	152.1	3.2	100.6		1.49
*D*-Glucitol	182.2	3.4	118.8		1.65
*D*-Mannitol	182.2	3.4	119.22	7.6 ± 4.8 × 10^−11^	0.906

^(a)^The values for glycerol, erythritol, xylitol and *D*-mannitol are from Kiyosawa [93]. Other values represent estimates of the present author.

^(b)^At infinite dilution at 25°C [94]. Values for ribitol and sorbitol are from Back et al. [95].

^(c)^Using the giant alga *Chara* cell membrane [96].

^(d)^The values of *K* are those of a correlating constant from the equation *a*_*w*_ = *x*_1_ exp(−*K* *x*_2_^2^), where *x*_1_ and *x*_2_ are molar fractions of water and solute, respectively, and *a*_*w*_ is water activity [97].

**Table 4:** Concentration of calcium (determined by means of atomic absorption spectrophotometry) in dental plaque of subjects who used products containing xylitol.

Study	Xylitol	Control or sucrose	Remarks
Chewing of xylitol gums (paraffin as control)	1.22 ± 0.45	0.78 ± 0.30	In *μ*g/mg fresh weight (*n* = 10–12; *P* < .01. Sorbitol gave similar results [98].

Chewing of xylitol gum (compared with sucrose gum and gum base)	3.7 ± 0.5	2.4 ± 0.2	In *μ*g/mg dry weight (*n* = 83). Gum base: 3.4 ± 0.7. Significance of differences was not given [99].

Rinsing with 0.4 M xylitol or sucrose solutions	0.90	0.67	In *μ*g/mg protein. Plaque pools from 11 subjects in both groups. 0.01 M Na cyclamate: 0.60 [100].

Xylitol or sorbitol chewing gum compared with no gum	1.77 ± 0.99	1.70 ± 1.33	In % dry weight in plaque. No gum: 1.24 ± 0.82%. For both polyols: *P* < .03 when compared with no gum. *n* = 25 [101].
